# Program

**DOI:** 10.1111/jdi.12669

**Published:** 2017-05-05

## Oral Sessions

**Friday, May 19 Room 5, Nagoya Congress Center [Bldg.1 4F Reception Hall (East)]**

**Table:** 

Oral Sessions‐1	13:30–14:30	
	Chairs Department of Endocrinology and Metabolism, Shanghai Jiao Tong University, Affiliated Sixth People's Hospital, Shanghai, China	**Weiping Jia**
	Department of Diabetes, Endocrinology and Nutrition, Kyoto University Graduate School of Medicine, Kyoto, Japan	**Nobuya Inagaki**
AI‐5‐1	Indium‐labeled Exendin probe enables to quantify beta cell mass non‐invasively with SPECT imaging	**Naotaka Fujita**
	Department of Diabetes, Endocrinology and Nutrition, Graduate School of Medicine, Kyoto University, Japan	
AI‐5‐2	Role of human urine‐derived stem cells in the treatment of diabetic mice	**Tianxue Zhao**
	Shanghai Diabetes Institute, Shanghai Jiao Tong University, Shanghai, People's Republic of China /Department of Endocrinology and Metabolism, Shanghai Jiao Tong University Affiliated Sixth People's Hospital, Shanghai, People's Republic of China	
AI‐5‐3	LAF237 Enhances Glucose Tolerance and Graft β‐Cell Mass in Diabetic Mice Transplanted with Sufficient Numbers of Islets	**Jyuhn‐Huarng Juang**
	Division of Endocrinology and Metabolism, Chang Gung University and Memorial Hospital, Taiwan	
AI‐5‐4	Suppression of ROS production by Exendin‐4 in PSC attenuates the high glucose‐induced islet fibrosis	**Jiwon Kim**
	Department of Endocrnology, The Catholic University of Korea	
AI‐5‐5	Impaired insulin secretion and severe glucose intolerance caused by deleting β‐cell specific KATP channels	**Okechi Oduori S.**
	Molecular and Metabolic Medicine, Kobe University Graduate School of Medicine, Japan	
AI‐5‐6	Effects of β‐cell function related genetic variants on predicting deterioration of glucose tolerance in the Chinese	**Jing Yan**
	Shanghai Diabetes Institute, Shanghai Jiao Tong university affiliated sixth people's hospital, China	

**Friday, May 19 Room 6, Nagoya Congress Center [Bldg.1 4F Reception Hall (West)]**

**Table:** 

Oral Sessions‐2	8:00–9:00	
	Chairs Eulji University Hospital, Seoul, Korea	**Hong Kyu Lee**
	Endocrine and Metabolism Department, Peking University People's Hospital, Beijing, China	**Linong Ji**
AI‐6‐1	Euglycemia by long‐term insulin pump therapy differentially improve C‐peptide or disposition index in type 2 diabetes	**Soobong Choi**
	Department of Internal Medicine, Konkuk University School of Medicine, Korea	
AI‐6‐2	Safety of metformin therapy and serum lactate level in T2DM living on an oxygen‐deficient plateau, Tibet, China	**Qian Ren**
	Department of Endocrinology and Metabolism, People's Hospital of Tibet Autonomous Region, China	
AI‐6‐3	Renoprotective effect of liraglutide in Japanese patients with type 2 diabetes enrolled in dialysis prevention program	**Yoshiyuki Hamamoto**
	Center for Diabetes, Endocrinology and Metabolism, Kansai Electric Power Hospital, Japan	
AI‐6‐4	Efficacy and Safety of a Fixed‐ratio Combination of Basal Insulin and GLP‐1 Receptor Agonist in T2DM Patients	**Xueying Gao**
	Department of Endocrinology and Metabolism, Peking University People's Hospital, China	
AI‐6‐5	SGLT2 inhibitor luseogliflozin use and dietary carbohydrate intake in Japanese individuals with type 2 diabetes	**Takuya Haraguchi**
	Center for Diabetes, Endocrinology and Metabolism, Kansai Electric Power Hospital, Osaka, Japan	
AI‐6‐6	Safety, tolerability and pharmacokinetics of Imeglimin in healthy Japanese subjects	**Sebastien Bolze**
	** **POXEL, France	

**Friday, May 19 Room 6, Nagoya Congress Center [Bldg.1 4F Reception Hall (West)]**

**Table:** 

Oral Sesions‐3	15:30–17:00	
	Chairs Taichung Veterans General Hospital, Taichung, Taiwan	**Wayne H‐H Sheu**
	Hallym University College of Medicine Seoul, Republic of Korea	**Doo‐Man Kim**
AI‐6‐7	Impaired glucagon‐like peptide 1 response is associated with metabolic syndrome in patients with type 2 diabetes	**Soyeon Yoo**
	Department of Internal Medicine, Jeju national University hospital, Korea	
AI‐6‐8	Neck circumference and waist circumference identify metabolic syndrome with similar efficacy	**Yuqi Luo**
	Department of Endocrinology and Metabolism, Shanghai Jiao Tong University Affiliated Sixth People's Hospital; Shanghai Clinical Center for Diabetes;Shanghai Key Clinical Center for Metabolic Disease; Shanghai Diabetes Institute; Shanghai Key Laboratory of Diabetes Mellitus, China	
AI‐6‐9	Relationship between vitamin D status, metabolic syndrome and incident diabetes: A prospective cohort study	**Eun Young Lee**
	Department of Internal Medicine, College of Medicine, The Catholic University of Korea	
AI‐6‐10	Xanthine oxidase activity in plasma reflects systemic insulin resistance in patients with T2DM and metabolic syndrome	**Sumito Sunagawa**
	Division of Endocrinology, Diabetes and Metabolism, Hematology, Rheumatology (Second Department of Internal Medicine), Graduate School of Medicine, University of the Ryukyus, Japan	
AI‐6‐11	Clinical prospects of altered mRNA expression levels of Matrix Metalloprotienases 1, 2 and 9 in metabolic syndrome.	**Suraj Yadav Singh**
	Department of VARD‐ Human Health, National Innovation Foundation, Ahmedabad, Gujarat, India/Department of Pharmacology, King George's Medical University, Lucknow, UP, India	
AI‐6‐12	Central obesity measured by Controlled Attenuation Parameter is more important factor to predict significant steatosis	**Jae Hyuk Lee**
	Department of Internal Medicine, SEONAM University college of Medicine, myongji hospital, Korea	
AI‐6‐13	Association between free triiodothyronine levels and nonalcoholic fatty liver disease in euthyroid Chinese	**Peizhu Chen**
	Department of Endocrinology and Metabolism, Shanghai Jiao Tong University Affiliated Sixth People's Hospital/Shanghai Clinical Center for Diabetes/Shanghai Diabetes Institute/Shanghai Key Laboratory of Diabetes Mellitus/Shanghai Key Clinical Center for Metabolic Disease, China	
AI‐6‐14	Lower 25‐hydroxy vitamin D level was associated with epicardial fat thickness and NAFLD disease in metabolic syndrome	**Eunheui Kim**
	Department of Internal Medicine, Pusan National University College of Medicine, Korea	
AI‐6‐15	Prevalence and risk factors for hyperlipidemia and hyperglycemia among Filipinos with HIV in a government treatment hub	**Marc Gregory Yu**
	Section of Endocrinology, Diabetes, and Metabolism, Department of Medicine, Philippine General Hospital, Philippines	

**Saturday, May 20 Room 5, Nagoya Congress Center [Bldg.1 4F Reception Hall (East)]**

**Table:** 

Oral Sessions‐4	15:10–16:10	
	Chairs Diabetes Center of the 306th Hospital, Beijing, China	**Zhangrong Xu**
	University of Malaya, Kuala Lumpur, Malaysia	**Dato'I kram Shah b Ismail**
AII‐5‐1	Withdrawn
AII‐5‐2	Effectiveness assessment for the treatment and management platform of obesity in Korean child and youth	**Sun‐Young Lim**
	Catholic Institute of U‐healthcare, The Catholic University of Korea	
AII‐5‐3	Mobile based nutritional management tool for pregnant women with diabetes mellitus in Korea	**Sun‐Young Lim**
	Catholic Institute of U‐healthcare, The Catholic University of Korea	
AII‐5‐4	Outcomes after hospitalization of diabetes in older patients received influenza vaccination	**Yi‐Chun Chou**
	Department of Physical Medicine and Rehabilitation, China Medical University Hospital, Taiwan	
AII‐5‐5	Severe hypoglycemia and risk of dementia in person with diabetes mellitus	**Kyoung Hwa Ha**
	Department of Endocrinology and Metabolism, Ajou University School of Medicine/Cardiovascular and Metabolic Disease Etiology Research Center, Ajou University School of Medicine, Korea	
AII‐5‐6	Glycemic variability and in‐hospital mortality among elderly patients in the ICU	**Anna Angelica Macalalad‐Josue**
	Section of Endocrinology, Diabetes, and Metabolism, Department of Medicine, Philippine General Hospital/Department of Clinical Epidemiology, College of Medicine, University of the Philippines Manila/Department of Medicine, The Medical City, Philippines	

**Saturday, May 20 Room 6, Nagoya Congress Center [Bldg.1 4F Reception Hall (West)]**

**Table:** 

Oral Sessions‐5	15:50–17:20	
	Chairs Department of Internal Medicine, National Taiwan University, Hospital Taipei, Taiwan	**Lee‐Ming Chuang**
	Yamaguchi University Graduate School of Medicine, Ube, Japan	**Yukio Tanizawa**
AII‐6‐7	Association Study of 14 Single Nucleotide Polymorphisms and Insulin Resistance in a Chinese Population	**Yeping Huang**
	Department of Endocrinology and Metabolism, Shanghai Jiao Tong University Affiliated Sixth People's Hospital, China	
AII‐6‐8	Role of Activin/FSTL3 in the glucose metabolism	**Yukiko Okazaki**
	Department of Diabetes and Metabolic Diseases, the University of Tokyo Hospital, Japan	
AII‐6‐9	Serum Aryl‐hydrocarbon Receptor Transactivation Assay Reveals Persistent Nature of Diabetogenic Chemicals	**Hong Kyu Lee**
	Department of Internal Medicine, Eulji University, Korea	
AII‐6‐10	Identifying a new pathway to regulate AMPK activity and autophagy under metabolic stress.	**Eijiro Yamada**
	Department of Medicine and Molecular Science, Japan	
AII‐6‐11	Enhanced expression of Survivin plays distinct roles in adipocyte homeostasis	**Liping Ju**
	Shanghai Institute for Diabetes/Shanghai Key Laboratory of Diabetes/Shanghai Clinical Medical Centre of Diabetes/Shanghai Key Clinical Centre of Metabolic Diseases/Shanghai Jiao‐Tong University Affiliated Sixth People's Hospital, China	
AII‐6‐12	SFPQ, a pre‐mRNA splicing factor interacts with phosphorylated HELZ2 and plays a novel role in adipocyte differentiation	**Akiko Toki Katano**
	Department of Medicine and Molecular Science, Gunma University Graduate School of Medicine, Japan	
AII‐6‐13	Imeglimin Increases Insulin Secretion in Response to Glucose as a Unique Mechanism of Action Depending on NAD Synthesis	**Sophie Hallakou‐Bozec**
	POXEL, France	
AII‐6‐14	Effect of liraglutide and monoclonal FGFR2‐IIIc antibody on the development of obesity and type 2 diabetes in mice	**Katsunori Nonogaki**
	Department of Diabetes Technology, Tohoku University Graduate School of Biomedical Engineering, Japan	
AII‐6‐15	A limonene‐derivative purified from Sudachi peel upregulates sirt1 and improves lipid/glucose metabolism in HFD‐fed mice	**Licht Miyamoto**
	Dept. Medical Pharmacology, Inst. of Biomedical Sciences, Graduate School of Tokushima University, Japan	

## Poster Sessions

**Friday, May 19 Poster Room, Nagoya Congress Center [Underground carpark]**

**Table:** 

Poster Sessions‐1	18:10–18:40	
	Chair Shanghai Jiaotong University Affiliated Sixth People's Hospital, China	**Cheng Hu**
AI‐P‐1	Association between haptoglobin genotype and diabetic macroangiopathies in Chinese patients with type 2 diabetes	**Shiyun Wang**
	Shanghai Diabetes Institute, Shanghai Key Laboratory of Diabetes Mellitus, Shanghai Clinical Center for Diabetes, Shanghai Jiao Tong University Affiliated Sixth People's Hospital, China	
AI‐P‐2	GCK‐MODY is not rare in a Chinese community population	**Yumin Ma**
	Beijing Pinggu Hospital, China	
AI‐P‐3	Predicting effects of G6PC2 on type 2 diabetes and impaired glucose regulation in a 9‐year prospective Chinese cohort	**Feng Jiang**
	Shanghai Diabetes Institute,Shanghai Jiao Tong University Affiliated Sixth People's Hospital/Shanghai Key Laboratory of Diabetes Mellitus/Shanghai Clinical Center for Diabetes, China	
AI‐P‐4	The association of variations in COX1 and COX2 with type 2 diabetes and glucose metabolism in Chinese Han populations	**Bo Xu**
	Department of Endocrinology, Shanghai Jiao Tong University Affiliated Sixth People's Hospital/Shanghai Diabetes Institute/Shanghai Key Laboratory of Diabetes Mellitus, China	
AI‐P‐5	Variations in KCNJ11 are associated with early‐onset type 2 diabetes mellitus in Chinese population	**Limei Liu**
	Department of Endocrinology & Metabolism, Shanghai Jiaotong University Affiliated Sixth People's Hospital, Shanghai Diabetes Institute, Shanghai Key Laboratory of Diabetes, China	
AI‐P‐6	Association of PAX4 polymorphism with diabetic retinopathy in Chinese population	**Yangyang Li**
	Department of Endocrine,Shanghai Jiao Tong University Affiliated Sixth People's Hospital, China	

**Friday, May 19 Poster Room, Nagoya Congress Center [Underground carpark]**

**Table:** 

Poster Sessions‐2	17:40–18:10	
	Chair Division of Rheumatology , Endocrinology and Nephrology, Hokkaido University Graduate School of Medicine, Sappono, Japan	**Akinobu Nakamura**
AI‐P‐7	Baseline level and change in serum albumin concentration and the risk of incident type 2 diabetes	**You Cheol Hwang**
	Department of Medicine, Kyung Hee University School of Medicine, Korea	
AI‐P‐8	Elevation in serum fibroblast growth factor 23 levels in normoglycemic first‐degree relatives of patients with diabetes	**Xiaojing Ma**
	Department of Endocrinology and Metabolism, Shanghai Jiao Tong University Affiliated Sixth People's Hospital; Shanghai Clinical Center for Diabetes; Shanghai Key Clinical Center for Metabolic Disease; Shanghai Diabetes Institute; Shanghai Key Laboratory of Diabetes Mellitus, China	
AI‐P‐9	Heavy alcohol drinking as a nonglycemic parameter to determine hemoglobin A1c in a Korean population	**Dong‐Jun Kim**
	Department of Internal Medicine, Inje University Collegel of Medicine Ilsanpaik Hospital, Korea	
AI‐P‐10	Comparisons between early onset and late onset patients of the glucose control in newly diagnosed T2DM in China	**Xiaoling Cai**
	Peking University People's Hospital, China	
AI‐P‐11	Withdrawn.	
AI‐P‐12	The association of CYP2C9 and CYP2J2 variants with type 2 diabetes risk in Chinese populations	**Tao Wang**
	Shanghai Diabetes Institute,Shanghai Jiao Tong University Affiliated Sixth People's Hospital/Shanghai Key Laboratory of Diabetes Mellitus,Shanghai Clinical Center for Diabetes, China	

**Friday, May 19 Poster Room, Nagoya Congress Center [Underground carpark]**

**Table:** 

Poster Sessions‐3	18:10–18:40	
	Chair Department of Endocrinology and Metabolism, Kangbuk Samsung Hospital, Sungkyunkwan University School of Medicine, Seoul, Korea	**Eun‐Jung Rhee**
AI‐P‐13	Retrospective review on the outpatients with type 1 diabetes mellitus at Chiba University Hospital.	**Jin Kumagai**
	Clinical Cell Biology and Medicine, Graduate School of Medicine, Chiba University/Department of Diabetes, Metabolism and Endocrinology, Chiba University Hospital Chiba, Japan	
AI‐P‐14	Effect of ethanolic leaf extract from Adenanthera pavonina (L) on blood sugar regulation in rats	**Ashoka Nuwarapakshage Sanjeewani**
	Department of Pharmacy, Faculty of Allied Health Sciences, General Sir John Kotelawala Defence University/Postgraduate institute of Agriculture, University of Peradeniya, Sri Lanka	
AI‐P‐15	Usage of CGM for management of glucocorticoid replacement therapy in adult patients with central hypoadrenalism	**Atsushi Ozawa**
	Department of Medicine and Molecular Science, Gunma University Graduate School of Medicine, Japan	
AI‐P‐16	Withdrawn.	
AI‐P‐17	Comparative Study on Prevalence and Cause of Hypoglycemic Attacks Observed at Emergency Room between 2010 and 2015	**Koichiro Nemoto**
	Department of Internal Medicine, Chiba Rosai Hospital, Japan	
AI‐P‐18	Factors complicating diabetes management for foreign patients with diabetes in Japan	**Miyako Kishimoto**
	Department of Internal Medicine, Sanno Hospital, Japan	

**Friday, May 19 Poster Room, Nagoya ter [Underground carpark]**

**Table:** 

Poster Sessions‐4	17:40–18:10	
	Chair Department of Internal Medicine, National Taiwan University Hospital, Seoul, Korea	**Hung‐Yuan Li**
AI‐P‐19	Usefulness of glucose parameters obtained from Oral glucose tolerance test (OGTT) in Korean subjects.	**Tae Sun Park**
	Department of Endocrinology and Metabolism, Chonbuk National University Medical School/Research Institute of Clinical Medicine, Chonbuk National University Hospital, Korea	
AI‐P‐20	Serum 1,5‐anhydroglucitol levels slightly increase after a glucose load	**Hang Su**
	Department of Endocrinology and Metabolism, Shanghai Jiao Tong University Affiliated Sixth People's Hospital; Shanghai Clinical Center for Diabetes; Shanghai Key Clinical Center for Metabolic Disease; Shanghai Diabetes Institute; Shanghai Key Laboratory of Diabetes Mellitus, China	
AI‐P‐21	Inverse association between fasting insulin levels and postprandial asprosin level changes in Type 2 diabetes patients	**Shinsuke Tokumoto**
	Centre for Diabetes and Endocrinology, Tazuke Kofukai Foundation, Medical Research Institute, Kitano Hospital, Japan	
AI‐P‐22	Response to 75 g glucose tolerance tests: comparison of type 2 diabetes and healthy control	**Saran Tserensodnom**
	Endomed clinic, Mongolia	
AI‐P‐23	HbA1C and mean glucose derived from continuous glucose monitoring assessment do not correlate in patients with HbA1c >8%	**Yuko Tagaya**
	Department of Medicine and Molecular Science Gunma University Graduate School of Medicine, Japan	
AI‐P‐24	Comparison of markers between metabolically obese but normal weight and metabolically healthy but obese in Korean adults	**Yang Ho Kang**
	Department of Internal Medicine, Pusan National University Yangsan Hospital, Pusan National University School of Medicine, Yangsan, Korea	

**Friday, May 19 Poster Room, Nagoya Congress Center [Underground carpark]**

**Table:** 

Poster Sessions‐5	18:10–18:40	
	Chair The First Department of Medicine, Wakayama Medical University, Wakayama, Japan	**Hiroto Furuta**
AI‐P‐25	The association of TCF7L2 genetic variants with diabetic retinopathy in Chinese population	**Xiao Wang**
	Shanghai Diabetes Institute, Shanghai Jiao Tong university affiliated sixth people's hospital, China	
AI‐P‐26	One Stop Service for survey of diabetic complications	**Chung Sen Chen**
	Department of Endocriniology and Metabolism, Taipei City Hospital, Taiwan	
AI‐P‐27	Association between FTO gene polymorphisms and osteoporosis in patients with T2DM	**Jianjin Guo**
	Department of Endocrinology, The second Affiliated Hospital of Shanxi Medical University, China	
AI‐P‐28	Risk of diabetes in patients of chronic obstructive pulmonary disease: a retrospective cohort study	**Chien‐Chang Liao**
	School of Medicine, Taipei Medical University, Taiwan	
AI‐P‐29	Effect of sleep disorders in patients with diabetes mellitus	**Sodai Kubota**
	Center for Diabetes, Endocrinology and Metabolism, Kansai Electric Power Hospital, Japan	
AI‐P‐30	Long term risk of stroke in type 2 diabetic patients with diabetic ketoacidosis	**Yu‐Li Chen**
	Division of Endocrinology and Metabolism, Department of Internal Medicine, Chi Mei Medical Center, Taiwan	

**Friday, May 19 Poster Room, Nagoya Congress Center [Underground carpark]**

**Table:** 

Poster Sessions‐6	17:40–18:05	
	Chair Department of Medical Nutritional Therapy, Chang Gung Memorial Hospital and Chang Gung University, Taoyuan City, Taiwan	**Yu‐Yao Huang**
AI‐P‐31	The impact of endothelial malfunction and postprandial hyperglycemia on adverse events among type 2 diabetic patients	**Tetsuhiko Sato**
	Division of Diabetes and Endocrinology, Masuko Memorial Hospital/Nagoya Daini Red Cross Hospital, Japan	
AI‐P‐32	The synergistic impact of apolipoprotein B/A‐1 and lipoprotein (a) on coronary artery calcification	**Kahui Park**
	Department of Internal Medicine, Gangnam Severance Hospital, Yonsei University College of Medicine, Korea	
AI‐P‐33	Establishment of a zebrafish system for diabetes complications by novel acceleration plethysmogram and electrocardiogram	**Taiji Ito**
	Department of Nutrition and Dietetics, Faculty of Family and Consumer Sciences, Kamakura Women's University, Japan	
AI‐P‐34	Fibroblast growth factor 23 strengthen the capacity of Framingham Risk Score to identify subclinical atherosclerosis	**Xiang Hu**
	Department of Endocrinology and Metabolism, Shanghai Jiao Tong University Affiliated Sixth People's Hospital; Shanghai Clinical Center for Diabetes; Shanghai Key Clinical Center for Metabolic Disease; Shanghai Diabetes Institute; Shanghai Key Laboratory of Diabetes Mellitus, China	
AI‐P‐35	Low‐frequency and very low‐intensity ultrasound decreases blood pressure in hypertensive subjects with type 2 diabetes	**Katsunori Nonogaki**
	Department of Diabetes Technology, Tohoku University Graduate School of Biomedical Engineering, Japan	

**Friday, May 19 Poster Room, Nagoya Congress Center [Underground carpark]**

**Table:** 

Poster Sessions‐7	18:10–18:40	
	Chair Department of Diabetes and Metabolic Diseases, Graduate School of Medicine, The University of Tokyo, Tokyo, Japan	**Ryo Suzuki**
AI‐P‐36	Negative Pressure Wound Therapy inhibit Inflammation in diabetic patients with foot ulcerations	**Rui He**
	Department of Endocrinology and Metabolism, Shanghai Jiaotong University Affiliated Sixth People's Hospital/Shanghai Key Laboratory of Diabetes/Shanghai Institute for Diabetes/Shanghai Clinical Medical Centre of Diabetes, China	
AI‐P‐37	Adjuvant effects of diabetes on hypertensive neuropathy in spontaneously hypertensive rats	**Hitoshi Nukada**
	The Nukada Institute for Medical & Biological Research, Japan/University of Otago, New Zealand	
AI‐P‐38	Factors associated with post discharge follow up among hospitalized patients with diabetic foot	**Diane Carla Bernardo**
	Section of Endocrinology, Diabetes and Metabolism, Department of Medicine, Philippine General Hospital, Philippines	
AI‐P‐39	DPN Check, a nerve conduction testing device, is useful as the Indicator for foot ulcer risk assessment	**Masuko Sumikawa**
	Department of Nursing, School of Health Sciences, Sapporo Medical University, Japan	
AI‐P‐40	Healing Progress of Diabetic Foot Ulcer	**Telmen Boldbaatar**
	Endomed clinic, Mongolia	
AI‐P‐41	Screening for Diabetic Autonomic Neuropathy using a Blood Pressure/Pulse Wave Measuring Apparatus	**Noriko Kato**
	Kato Clinic of Internal Medicine, Japan	

**Friday, May 19 Poster Room, Nagoya Congress Center [Underground carpark]**

**Table:** 

Poster Sessions‐8	17:40–18:10	
	Chair Kanazawa Medical University, Kanazawa, Japan	**Keizo Kanasaki**
AI‐P‐42	Comparison of uric acid levels and mortality and risk of renal‐replacement therapy in DM and non‐DM CKD patients	**Chun‐Yi Wu**
	Division of Nephrology, Department of Medicine, Taichung Veterans General Hospital, Taiwan	
AI‐P‐43	Hemorheological approach for screening diabetic nephropathy in type 2 diabetes	**Junghye Kim**
	Department of Internal Medicine, Gangnam Severance Hospital, Seoul, Korea	
AI‐P‐44	Arg913Gln of SLC12A3 gene promotes development and progress of DN‐ESRD in Chinese type 2 diabetes mellitus	**Limei Liu**
	Department of Endocrinology & Metabolism, Shanghai Jiaotong University Affiliated Sixth People's Hospital, Shanghai Diabetes Institute, Shanghai Key Laboratory of Diabetes, China	
AI‐P‐45	Aberrant STRA6 and CRBP1 signal pathway is involved in development of diabetic nephropathy via mitochondrial dysfunction	**Shyi Shin Jang**
	Department of Internal Medicine, Kaohsiung Medical University Hospital/Graduate Institute of Medicine, Kaohsiung Medical University, Taiwan	
AI‐P‐46	The association of a genetic variant in SCAF8‐CNKSR3 with diabetic microvascular disease in a Chinese population	**Li Jin**
	Shanghai diabetes institute, China	
AI‐P‐47	Abnormal thermal thresholds of lower extremities is significantly associated with low TBI in type 2 diabetic patients	**Yi‐Jing Sheen**
	Division of Endocrinology and Metabolism, Department of Internal Medicine, Taichung Hospital, Ministry of Health and Welfare/Department of Public Health, China Medical University, Taiwan	

**Saturday, May 20 Poster Room, Nagoya Congress Center [Underground carpark]**

**Table:** 

Poster Sessions‐9	16:20–16:45	
	Chair Seoul St. Mary's Hospital, The Catholic University Medical College, Seoul, Korea	**Kun‐Ho Yoon**
AII‐P‐1	Analysis of the Syndecan‐4 gene expression control mechanism in MIN6 cell	**Iwao Takahashi**
	Department of Medical Biochemistry, School of Pharmacy, Iwate Medical University, Japan	
AII‐P‐2	Differentiation of human induced pluripotent stem cells into insulin‐producing cells	**Min Jung Kim**
	Department of Endocrinology, The Catholic University of Korea	
AII‐P‐3	Generation of the beta‐like cells differentiated into the Insulin‐producing cells from Mouse small intestine.	**So Hyun Lee**
	Department of Endocrinology and metabolism, The Catholic University of Korea	
AII‐P‐4	Chronic Arsenic Exposure Impairs Pancreatic β‐cell Function	**Christopher Carmean M.**
	Division of Molecular and Metabolic Medicine, Kobe University, Japan	
AII‐P‐5	Small molecules exert anti‐apoptotic effect on ICAs	**Bhawna Chandravanshi**
	School of Regenerative Medicine, Manipal University, India	

**Saturday, May 20 Poster Room, Nagoya Congress Center [Underground carpark]**

**Table:** 

Poster Sessions‐10	16:50–17:15	
	Chair Department of Diabetes and Metabolic Diseases, Graduate School of Medicine, The University of Tokyo, Tokyo, Japan	**Hironori Waki**
AII‐P‐6	Whole exome sequencing identifies a novel INS mutation as a hot spot mutation of Maturity‐onset Diabetes of the Young	**Cheng Hu**
	Shanghai Diabetes Institute, Shanghai Jiao Tong University Affiliated Sixth People's Hospital/Shanghai Key Laboratory of Diabetes Mellitus/Shanghai Clinical Center for Diabetes, China	
AII‐P‐7	ATP and Ca^2+^ dynamics in pancreatic b‐cells: Ex vivo analysis using ATP biosensor GO‐ATeam1	**Ryota Usui**
	Department of Diabetes, Endocrinology and Nutrition, Graduate School of Medicine Kyoto University, Japan	
AII‐P‐8	GPR120 mediated pdx1 signaling protects against palmitic acid‐induced pancreatic beta‐cell dysfunction	**Yi Wang**
	School of Biomedical sciences, The Chinese University of HongKong	
AII‐P‐9	Sphingosine kinase 1‐interacting protein (SKIP) is a novel regulator of glucose‐stimulated insulin secretion	**Yu Wang**
	Department of Diabetes, Endocrinology and Nutrition, Graduate School of Medicine, Kyoto University, Japan	
AII‐P‐10	Association of Akt activation and adiponectin‐enhanced glucose stimulated insulin secretion in beta‐cells*	**Hui Zhu**
	Shanghai Diabetes Institute, Shanghai Jiao Tong University, China	

**Saturday, May 20 Poster Room, Nagoya Congress Center [Underground carpark]**

**Table:** 

Poster Sessions‐11	16:20–16:45	
	Chair Department of Diabetes, Endocrinology and Nutrition, Graduate School of Medicine, Kyoto University, Kyoto, Japan	**Norio Harada**
AII‐P‐11	Protection Against High‐fat Diet Induced Obesity in Helz2‐Deficient Mice by Enhancing Hepatic Leptin Sensitivity	**Satoshi Yoshino**
	Department of Medicine and Molecular Science, Gunma University Graduate School of Medicine, Japan	
AII‐P‐12	Association of circulating miR‐29 with non‐alcoholic fatty liver disease	**Zhen He**
	Department of Endocrinology and Metabolism, Shanghai Jiao Tong University affiliated Sixth Poeple's Hospital, China	
AII‐P‐13	Depletion of sphingosine kinase 1‐interacting protein (SKIP) induces metabolically healthy obesity	**Yanyan Liu**
	Department of Diabetes, Endocrinology and Nutrition, Graduate School of Medicine, Kyoto University, Japan	
AII‐P‐14	The Drosophila RNF34 modulates lipid metabolism and exercise ability	**Ping Wei**
	Department of Endocrinology and Metabolism, Shanghai Jiao Tong University Affiliated Sixth People's Hospital, Shanghai Diabetes Institute, Shanghai Clinical Center for Diabetes, Shanghai, China	
AII‐P‐15	Analysis of age‐dependent leptin‐signaling in the leptin receptor deficient mice	**Pulong Pralampita Wijang**
	Department of Hygiene and Health Promotion Medicine, Graduate School of Medical and Dental Sciences, Kagoshima University, Japan	

**Saturday, May 20 Poster Room, Nagoya Congress Center [Underground carpark]**

**Table:** 

Poster Sessions‐12	16:50–17:20	
	Chair Kyoto University Graduate School of Medicine/Kansai Electric Power Medical Research Institute, Kyoto and Osaka, Japan	**Daisuke Yabe**
AII‐P‐16	Follistatin‐like 1 Protects Cardiomyoblasts from Injury Induced by Hyperglycemia	**Weiqian Chen**
	Institute for Cardiovascular Science of Soochow University, China	
AII‐P‐17	Anti‐diabetic effects of erythritol and associated molecular mechanisms investigated at ex vivo and type 2 diabetic rats	**Md. Shahidul Islam**
	Department of Biochemistry, University of KwaZulu‐Natal (Westville Campus), South Africa	
AII‐P‐18	Senescent CD8^+^ T Cells is Functionally Activated in Subjects with Prediabetes	**Hyon‐Seung Yi**
	Department of Internal Medicine, Chungnam National University School of Medicine, Korea	
AII‐P‐19	T cells analysis of Oral combined antibiotic treatment in STZ‐induced Diabetes Mice Model	**Zan‐chao Liu**
	The Diabetes Research Institute of The Shijiazhuang Second Hospital, China	
AII‐P‐20	Apps ameliorates diabetic nephropathy progression by transcriptional repression of TGFβ1 through interaction with Nrf2	**Pan Gao**
	Department of Endocrine, Shanghai Jiaotong University Affiliated to Sixth People's Hospital, Shanghai, China	
AII‐P‐21	Perilipin 1 in macrophage protects the progression of atherosclerosis via the change of macrophage polarity	**Kohei Yamamoto**
	Division of Rheumatology, Endocrinology and Nephrology at the Graduate School of Medicine, Hokkaido University, Japan	

**Saturday, May 20 Poster Room, Nagoya Congress Center [Underground carpark]**

**Table:** 

Poster Sessions‐13	16:20–16:50	
	Chair Diabetes Philippines, Philippines	**Tommy S. Ty Willing**
AII‐P‐22	Short‐ and long‐term (6‐ and 24‐month) effects of exenatide in patients with type 2 diabetes	**Osamu Ebisui**
	Department of Diabetes and Endocrinology, Ehime Prefectural Central Hospital, Japan	
AII‐P‐23	Retrospective analysis between the efficacy of combination therapy of liraglutide and basal insulin and β‐cell function	**Yui Sakuramachi**
	Center for Diabetes, Endocrinology, and Metabolism, Kansai Electric Power Hospital, Japan	
AII‐P‐24	Outcomes of efficacy and safety with alogliptin in eGFR study : results of 156 weeks follow up	**Takanori Hyo**
	Center for Diabetes, Endocrinology and Metabolism, Kansai Electric Power Hospital, Japan	
AII‐P‐25	Efficacy and safety of evoglipitin monotherapy in patients with type 2 diabetes	**Doo‐Man Kim**
	Department of Internal Medicine, Hallym University College of Medicine, Korea	
AII‐P‐26	Identical Hypoglyceamic Effect of Teneligliptin with Linagliptin switched from sitagliptin in Type 2 Diabetes with CKD	**Yoshiharu Wada**
	Center for Diabetes and Endocrinology, Tazuke Kofukai Foundation Medical Research Institute Kitano Hospital, Japan	
AII‐P‐27	Dipeptidyl peptidase‐4 inhibitor‐induced bullous pemphigoid: Report of three cases.	**Satoshi Yoshiji**
	Department of Diabetes, Endocrinology and Nutrition, Kyoto University Graduate School of Medicine, Japan	

**Saturday, May 20 Poster Room, Nagoya Congress Center [Underground carpark]**

**Table:** 

Poster Sessions‐14	16:50–17:10	
	Chair Asfendiyarov Kazakh National Medical University, Kazakhstan	**Zhanay A. Akanov**
AII‐P‐28	SGLT2 inhibitor and DPP4 inhibitor co‐administration in Thai diabetic patients	**Nalin Yenseung**
	Diabetes and Thyroid Center, Theptarin Hospital, Thailand	
AII‐P‐29	The relationship between morning spot urinary glucose parametersand HbA1c in patients with T2D after taking SGLT2i	**Byung Wan Lee**
	Yonsei University, Korea	
AII‐P‐30	Type2 Diabetes complicating with NASH can be effectively controlled by Laennec^®^ through normalizing Iron Metabolism	**Yuki Hamada**
	Hamada Clinic for Gastroenterology, Japan	
AII‐P‐31	Clinical Characteristics according to the use of Metformin in Korean Patients with Type 2 Diabetes Mellitus	**In Gyoon Ha**
	Department of Endocrinology and Metabolism, Kyung Hee University School of Medicine, Korea	

**Saturday, May 20 Poster Room, Nagoya Congress Center [Underground carpark]**

**Table:** 

Poster Sessions‐15	16:20–16:55	
	Chair Department of Medicine and Therapeutics, The Chinese University of Hong Kong, Hong Kong, China	**Ronald Ma**
AII‐P‐32	The Effectiveness of Continuous Glucose Monitoring (CGM) in self‐management of people with diabetes	**Michiko Gushiken**
	Department of Global health, Ryukyu University School of Medicine, Japan	
AII‐P‐33	Evaluation psychometric properties of patients Type 2 diabetes	**Bulgan Munkhtur**
	Mongolian National University of Medical Sciences/School of Nursing, Mongolian National University of Medical Sciences, Mongolia	
AII‐P‐34	Happiness among patients with diabetes is affected by diabetes control and support	**Fumika Mano**
	Department of Diabetes, Endocrinology and Nutrition, Graduate School of Medicine, Kyoto University, Japan	
AII‐P‐35	Impact of individual and spontaneous goal setting in life for patients with diabetes mellitus	**Shinji Ueno**
	Centor for Diabetes, Endocrinology and Metabolisum Kansai Electric Power Hospital, Japan	
AII‐P‐36	The glycemic control and behavior change after decrease of diabetic education frequency	**Ying‐Chuen Lai**
	Department of Internal Medicine, National Taiwan University Hospital/Graduate Institute of Clinical Medicine, National Taiwan University, Taiwan	
AII‐P‐37	Nurses' Implementation and Opinion of Assessment of Oral Health Behavior in Patients with Diabetes	**Yumi Kuwamura**
	Department of Nursing, Institute of Biomedical Sciences, Tokushima University Graduate School, Japan	
AII‐P‐38	Characteristics and efficacy of our diabetes support team: a physician's viewpoint	**Yu Kouchi**
	Diabetes Support Team, Orimoto Hospital, Japan	

**Saturday, May 20 Poster Room, Nagoya Congress Center [Underground carpark]**

**Table:** 

Poster Sessions‐16	16:50–17:15	
	Chair Fukuoka University, School of Medicine, Department of Endocrinology and Diabetes Mellitus, Fukuoka, Japan	**Takashi Nomiyama**
AII‐P‐39	Hyperglycemia from a rare case of ectopic ACTH‐secreting medullary thyroid carcinoma	**Antonio Jr. Faltado Lumbera**
	Section of Endocrinology, Diabetes and Metabolism, Department of Medicine, University of the Philippines ‐ Philippine General Hospital, Philippines	
AII‐P‐40	Newly identified mutation in 5′UTR of HNF1A in a case of MODY3	**Yukari Kitamura**
	Internal Medicine, Fujisawa Shonandai Hospital, Japan	
AII‐P‐41	Persistent Hypoglycemia in a Patient with Giant Phyllodes Tumor Successfully Treated with Dexamethasone and Surgery	**Jose Paolo Panuda Pacaira**
	Section of Endocrinology, Diabetes and Metabolism, University of the Philippines‐Philippine General Hospital, Philippines	
AII‐P‐42	Sodium glucose cotransporter 2 combined with Cetuximab attenuated multiple liver metastatic colon cancer	**Shuichi Okada**
	Division of Endocrinology and Diabetes Gunma University Hospital, Japan	
AII‐P‐43	Pancreatic tuberculosis mimicking pancreatic malignancy in a 33‐year old male with diabetes	**Annie Jane Sarmiento**
	Section of Endocrinology, Diabetes and Metabolism, Department of Medicine, University of the Philippines ‐ Philippine General Hospital, Philippines	

**Saturday, May 20 Poster Room, Nagoya Congress Center [Underground carpark]**

**Table:** 

Poster Sessions‐17	16:20–16:50	
	Chair Nagoya University, Nagoya, Japan	**Yusuke Seino**
AII‐P‐44	Relationship between Serum Lipocalin 2 Levels and Visceral Adiposity in Chinese Men	**Yuqian Bao**
	Department of Endocrinology and Metabolism, Shanghai Jiao Tong University Affiliated Sixth People‘s Hospital; Shanghai Clinical Center for Diabetes; Shanghai Key Clinical Center for Metabolic Disease; Shanghai Diabetes Institute; Shanghai Key Laboratory of Diabetes Mellitus, China	
AII‐P‐45	Bile acid profiles in gestational diabetes mellitus	**Fang Liu**
	Department of Endocrinology & Metabolism, Shanghai Jiao‐Tong University Affiliated Sixth Peoples Hospital, China	
AII‐P‐46	Japanese BMI Cut‐off Points to Screen for Type 2 Diabetes: A 5‐year Follow‐up Study.	**Yasuyo Nakajima**
	Department of Medicine and Molecular Science, Gunma University Graduate School of Medicine, Japan	
AII‐P‐47	Association of osteocalcin with glucose metabolism in the Chinese population	**Dandan Yan**
	Department of Endocrinology, Shanghai Jiao Tong University Affiliated Six People's Hospital, China	
AII‐P‐48	Lower Hedonic Hunger is Associated with Good Glycemic Control in Obese Chinese Patients with Type 2 Diabetes	**Lorena Tsui Fun Cheung**
	Department of Medicine and Therapeutics, The Chinese University of Hong Kong, Prince of Wales Hospital, China	
AII‐P‐49	Triglyceride‐to‐HDL Cholesterol Ratio is More Accurate in Detecting Metabolic Syndrome among middle‐aged Chinese	**Yuexing Liu**
	Shanghai Diabetes Institute, Shanghai Jiaotong University Affiliated Sixth People's Hospital, Shanghai, China	

